# Assessing the Knowledge and Practices of Burn First Aid in Al-Baha Region, Saudi Arabia

**DOI:** 10.7759/cureus.42879

**Published:** 2023-08-02

**Authors:** Mohammed N Asiri, Mohammed Y Bakhiet, Mujib M Alzahrani, Hashim A Alghamdi, Marwan N Alsaedi, Abdulmalek A Alshomrani, Anwar A Alghamdi, Tahani M Alghamdi

**Affiliations:** 1 Department of Plastic Surgery and Hand Surgery, Faculty of Medicine, Al-Baha University, Al-Baha, SAU; 2 Department of Surgery, Faculty of Medicine, Al-Baha University, Al-Baha, SAU; 3 Department of Surgery, Faculty of Medicine, University of Kordofan, Elobeid, SDN; 4 Faculty of Medicine, Al-Baha University, Al-Baha, SAU

**Keywords:** al-baha, awareness, knowledge, first aid, assessing, burn

## Abstract

Background

Burns are a prevalent type of injury that can result in substantial morbidity and mortality. Burn first aid knowledge is essential for reducing its complications and improving outcomes. However, evidence of the amount of burn first aid knowledge among the general population in many nations is sparse.

Methodology

A cross-sectional survey was conducted among 346 persons over the age of 18 from the Al-Baha region of Saudi Arabia. The participants’ knowledge of first aid for burns, including the appropriate steps to take in the event of a burn, the types of burns, and the proper application of burn dressings was assessed using a structured questionnaire.

Results

The majority of participants (73.6%) had inadequate knowledge of first aid for burns, while only 26.4% had adequate knowledge. The most common misconception among participants was the use of toothpaste, honey, or ice for burn treatment, which is not recommended. Additionally, hot water was the main cause of burning in this study.

Conclusions

This study underscores the necessity for targeted education and awareness-raising activities to improve the general population’s knowledge and habits about first aid for burns. Such programs can be tailored to clarify myths and misunderstandings regarding burn treatment and encourage evidence-based strategies for preventing and treating burns.

## Introduction

Burn injuries are a serious public health issue worldwide, with a wide range of repercussions including physical, functional, occupational, and cosmetic damage, as well as psychological anguish [1,2]. Burns are one of the top causes of death and disability in many countries [3,4]. Burn injuries are a major health concern in Saudi Arabia, with a high incidence rate observed in several regions of the country [5,6]. Despite advances in surgical and medical care for burns, mortality rates among burn victims remain high, particularly in developing nations. Therefore, it is necessary to have a good understanding of burn first aid to reduce the severity of the damage [7].

In a recent study conducted in Saudi Arabia, both medical and non-medical students were shown to have an inadequate understanding of burn first aid, with only around half of the participants able to correctly identify first aid treatments [8]. Surprisingly, the responses of medical students were not more accurate than those of non-medical students. These results show that the general public in Saudi Arabia may have an insufficient understanding of burn first aid and that education and public information resources can be useful in resolving this issue.

Multiple variables, including smoking, electrical appliances, cooking, water heaters, and chemical items, contribute to burns in the home, where they occur most frequently [1,9].

Initial management of burns, which includes first aid procedures at the site of the burn and therapy before transport to a burns center, is crucial in the treatment of burns. In the event of a burn injury, the degree of damage, discomfort, and problems can be mitigated with the application of simple and proper early therapy [8]. The majority of participants in a study conducted in the eastern province of Saudi Arabia utilized cold water as the most common first aid treatment for burn injuries, which is considered reasonable [10]. Therefore, it is hoped that the investigated region has a high degree of burn first aid knowledge.

When administering burn first aid, it is essential to stop the burn process, use tap water immediately for 20 minutes, remove clothing and jewelry from the wound, and cover it with a sterile dressing. However, incorrect burn first aid procedures might have negative consequences [1,6,8].

Currently, there is a lack of published studies on the knowledge and practices of burn first aid management in the Al-Baha region of Saudi Arabia, despite the region’s relatively high incidence of burn injuries. Therefore, this study aims to evaluate the public’s understanding of burn first aid care in the Al-Baha region of Saudi Arabia and to suggest areas for improvement. Specifically, the study aims to assess the degree of knowledge and awareness of burn first aid management among the general public in the Al-Baha region, as well as to identify the most often employed burn first aid management techniques. The findings of this study will inform the implementation of focused training programs and public awareness campaigns to enhance the understanding and practices of burn first aid management in Al Baha, Saudi Arabia, and, ultimately, the quality of care for burn victims.

## Materials and methods

Study design

This was a cross-sectional, community-based survey conducted in the Al Baha region of Saudi Arabia from March 20, 2023, to July 20, 2023. The study included both urban and rural areas.

Inclusion and exclusion criteria

The study included participants who were 18 years of age or older and who agreed to participate in the survey. Participants under the age of 18 were excluded from the study.

Sample size

The research focused on adults who met certain criteria and lived in the Al-Baha region. To determine the appropriate sample size, the researchers used the Raosoft sample size calculator with a 5% margin of error, 95% confidence interval, 50% response distribution, and a population size of 487,108 according to the General Authority for Statistics. Based on these factors, the researchers determined that a sample size of 346 participants would be sufficient to ensure the generalizability of the screening. The Raosoft sample size calculator uses a formula that takes into account the level of confidence, sample proportion, margin of error, and population size. The study used a non-probability convenience sampling technique to collect the results.

Data collection

Data were collected using a validated questionnaire in Google Forms. The questionnaire was available in both Arabic and English. All participants were informed in detail about the study aims, as well as data confidentiality. The questionnaire required consent from participants to participate in this study. Participants who agreed to participate in the study were asked to complete the questionnaire. The questionnaire was distributed through social media platforms, community centers, and hospitals to all Al Baha area provinces. The response was 500 participants, and 346 participants were selected according to the inclusion criteria.

Data analysis

Data were collected and analyzed using the SPSS statistical software (IBM Corp., Armonk, NY, USA). Descriptive statistics were used to summarize the data. The chi-square test was used to determine the association between different variables.

Questionnaire

The questionnaire included questions related to personal information, exposure to burns, knowledge of first aid for burns, and the source of information. The questionnaire was pretested on a small sample of participants to ensure the clarity and reliability of the questions.

Ethical considerations

Ethical approval was obtained from the Al-Baha University Medical School Ethical Committee (approval number: REC/SUR/BU-FM/2023/7). Participants were informed of the study aims and the confidentiality of their data. They were given the option to withdraw from the study at any time without any reprisals. The anticipated benefits of the study were explained to the participants.

Data management

Data were stored securely in password-protected files and were accessible only to the research team. The data will be kept for five years after the study’s completion and then destroyed.

## Results

A total of 346 participants were included in the study, with the majority being female (62.1%). The participants’ ages ranged from 18 to over 60 years, with the majority between the ages of 18 and 30 years (57.8%). The majority of participants were university-educated (70.8%), and the majority of them owned their own property (78.0%). The majority of participants were from Baha (30.0%), followed by Baljurashi (28.3%). The socioeconomic position of the participants was classified into three categories, namely, low, intermediate, and high, with the intermediate category containing the largest proportion of participants (33.8%) (Table 1).

**Table 1:** Demographic factors of the participants.

		Count	%
Age (year)	18–30	200	57.8%
	31–45	93	26.9%
	46–60	47	13.6%
	>60	6	1.7%
Gender	Male	131	37.9%
	Female	215	62.1%
Residence	Baha	135	39.0%
	Al Makhwah	12	3.5%
	Qilwah	26	7.5%
	Al Qara	10	2.9%
	Almandaq	6	1.7%
	Baljurashi	98	28.3%
	El Shaara	0	0.0%
	Bani Hasan	9	2.6%
	Other	50	14.5%
Educational level	Primary	4	1.2%
	Intermediate	14	4.0%
	Secondary	83	24.0%
	University	245	70.8%
Socioeconomic status	Low	125	36.1%
	Intermediate	117	33.8%
	High	104	30.1%
Housing status	Property	270	78.0%
	Rent	76	22.0%

Of the 346 participants, 71.4% reported that they or a member of their family had been burned at any degree, while 28.6% reported no history of burns. Among those who reported being burned, hot water was the most common cause of burn (28.3%), followed by hot surfaces (19.9%), and incendiary chemicals (10.1%). Regarding the place of burn, 63% of burns occurred inside the house, while 8.1% occurred outside the house. Among those who reported being burned, 44.5% had to go to the hospital for treatment, while 25.7% did not require hospitalization. When asked about the degree of burn, 25.1% reported first-degree burns, 21.4% reported second-degree burns, and 6.1% reported third-degree burns. A significant proportion (18.5%) reported not knowing the degree of the burn they had experienced. Regarding preparedness, 63.9% of participants reported having a first-aid kit at home, while 36.1% did not. Only 42.2% reported having a fire extinguisher at home, while 57.8% did not (Table 2).

**Table 2:** Exposure to burns.

		Count	%
Have you ever been burned, or a member of your family, of any degree?	Yes	247	71.4%
	No	99	28.6%
Cause of burn	Never being burned	104	30.1%
	Flame	33	9.5%
	Incendiary chemical	35	10.1%
	Hot water	98	28.3%
	Hot surface	69	19.9%
	Other	7	2.0%
Place of burn	Never being burned	100	28.9%
	Inside the house	218	63.0%
	Outside the house	28	8.1%
Did you need to go to the hospital?	Never being burned	103	29.8%
	Yes	154	44.5%
	No	89	25.7%
Degree of burn	Never being burned	100	28.9%
	First degree	87	25.1%
	Second degree	74	21.4%
	Third degree	21	6.1%
	Do not know	64	18.5%
Do you have a first-aid kit at home?	Yes	221	63.9%
	No	125	36.1%
Do you have a fire extinguisher at home?	Yes	146	42.2%
	No	200	57.8%

The majority of participants (43.1%) reported that the first step in the burning process is to stop the flame, while 54% of participants reported that the best method for burn first-aid management is to irrigate with tap water. Most participants (56.1%) reported that irrigation should be done immediately after the burn and for 10 minutes (41.3%). Only 37% of participants reported that it is important to cover the burn, and among those who said yes, the most common way to cover the burn was using a clean cloth (15.3%). A significant proportion of participants (73.4%) believed that it is necessary to seek medical advice and go to the hospital, while 19.7% believed that they do not need to do so. When asked about the medication they used for burn wounds to heal, 50% of participants believed that superficial wounds heal by local medication, while 23.7% believed that all burn wounds heal by using local medication (Table 3).

**Table 3:** Knowledge of first aid for burns.

		Count	%
What do you do first in the burning process?	Stop the flame	149	43.1%
	Remove clothes	99	28.6%
	Escape from the area	90	26.0%
	Do nothing	8	2.3%
What is the best method for burn first-aid management?	Irrigate with tap water	187	54.0%
	Putting ice on the place of burning	39	11.3%
	Putting honey	51	14.7%
	Putting toothpaste	21	6.1%
	Covering with a cloth	4	1.2%
	Putting ointment for burning	5	1.4%
	Other	2	0.6%
	I do not know	37	10.7%
Best time for irrigation	10 minutes	143	41.3%
	10–15 minutes	81	23.4%
	15–30 minutes	22	6.4%
	I do not know	100	28.9%
Best time for irrigation	Immediately	194	56.1%
	After 5 minutes	44	12.7%
	Until the burning zone cools	42	12.1%
	I don’t know	66	19.1%
Drinking enough fluids after any burn	Useful and necessary	204	59.0%
	Harmful and not recommended	30	8.7%
	I do not know	112	32.4%
Is it important to cover the burn?	Yes	128	37.0%
	No	157	45.4%
	I do not know	61	17.6%
If yes, how do you cover it?	No	178	51.4%
	Blanket	45	13.0%
	Clean clothes	53	15.3%
	Other	13	3.8%
	I do not know	57	16.5%
Is it important to keep the body warm?	Yes	104	30.1%
	No	113	32.7%
	I do not know	129	37.3%
In your opinion, do you need to seek medical advice and go to the hospital?	Yes	254	73.4%
	No	68	19.7%
	I do not know	24	6.9%
Do all burn wounds heal by using local medication?	Yes	82	23.7%
	No	91	26.3%
	Superficial	173	50.0%

**Figure 1:**
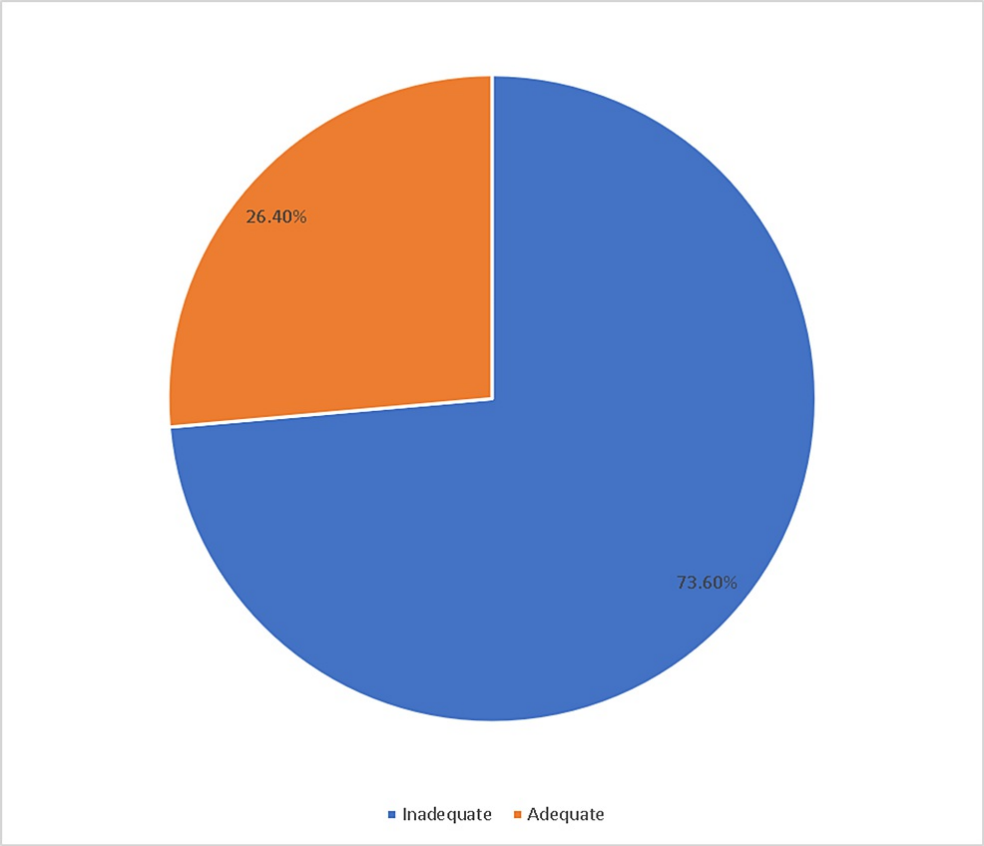
Participants’ level of knowledge about first aid for burns.

As shown in Figure 1, the majority of participants (73.6%) had inadequate knowledge of first aid for burns, while only 26.4% had adequate knowledge.

As shown in Table 4, there was no significant relationship between knowledge of first aid for burns and age, gender, residence, educational level, socioeconomic status, or history of burns. However, there was no significant relationship between housing status and knowledge of first aid for burns, with participants who owned property having a lower percentage of adequate knowledge (24.1%) compared to those who rented (36.8%).

**Table 4:** Relationship between knowledge and demographic factors.

		Knowledge				
		Inadequate		Adequate		P-value
		Count	%	Count	N %	
Age (year)	18–30	145	72.5%	55	27.5%	0.855
	31–45	70	75.3%	23	24.7%	
	46–60	33	70.2%	14	29.8%	
	>60	5	83.3%	1	16.7%	
Gender	Male	101	77.1%	30	22.9%	0.193
	Female	152	70.7%	63	29.3%	
Residence	Baha	102	75.6%	33	24.4%	0.829
	Al Makhwah	8	66.7%	4	33.3%	
	Qilwah	20	76.9%	6	23.1%	
	Al Qara	9	90.0%	1	10.0%	
	Almandaq	4	66.7%	2	33.3%	
	Baljurashi	69	70.4%	29	29.6%	
	El Shoora	0	0.0%	0	0.0%	
	Bani Hasan	7	77.8%	2	22.2%	
	Other	34	68.0%	16	32.0%	
Educational level	Primary	3	75.0%	1	25.0%	0.965
	Intermediate	10	71.4%	4	28.6%	
	Secondary	59	71.1%	24	28.9%	
	University	181	73.9%	64	26.1%	
Socioeconomic status	Low	92	73.6%	33	26.4%	0.986
	Intermediate	85	72.6%	32	27.4%	
	High	76	73.1%	28	26.9%	
Housing status	Property	205	75.9%	65	24.1%	0.027
	Rent	48	63.2%	28	36.8%	
Have you ever been burned, or a member of your family, of any degree?	Yes	179	72.5%	68	27.5%	0.666
	No	74	74.7%	25	25.3%	

**Figure 2:**
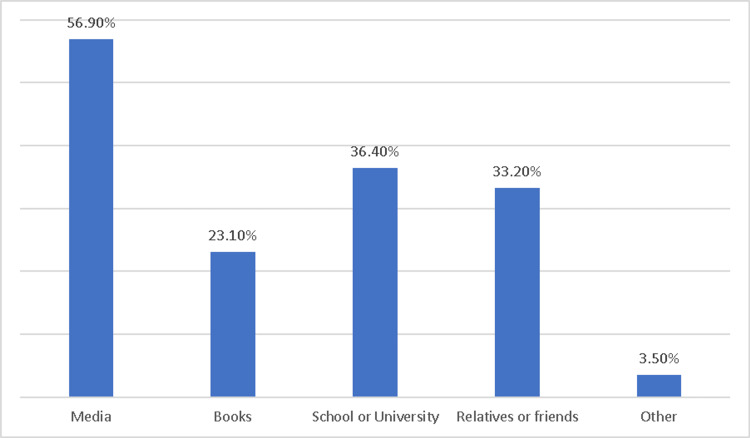
Source of knowledge about first aid for burns.

According to Figure 2, the most common source of knowledge for first aid for burns was media (56.9%), followed by school or university (36.4%), relatives or friends (33.2%), and books (23.1%). Only a small percentage of participants reported other sources of knowledge (3.5%).

## Discussion

The findings of this study provide valuable insights into the knowledge and practices of first aid for burns among a sample of Saudi Arabians in the Al-Baha region. The findings indicate a significant need for education and awareness-raising programs to improve the community’s knowledge and practices regarding burn first-aid management.

The majority of participants (73.6%) possessed inadequate knowledge of first aid for burns, while only 26.4% possessed adequate knowledge. Multiple studies have investigated the knowledge and practices of various populations regarding burn first aid. Variable results have been, with some studies reporting similar results to the present study while others reporting different results. For instance, according to a study conducted in Pakistan, only 15% of participants possessed adequate knowledge of first aid for burns, while the majority possessed inadequate knowledge [11]. According to a separate study conducted in Iran, 45% of participants had inadequate knowledge of burn first aid [12]. In contrast, some studies found that participants have greater knowledge of first aid for burns. In a previous study conducted in Saudi Arabia, the authors showed that the majority of the respondents (63%) had a satisfactory response to self-aid alone at home [10]. Moreover, 64% of participants in a separate study conducted in Malaysia possessed adequate knowledge of first aid for burns [13]. This highlights the urgent need for targeted education and awareness programs to improve the community’s burn first-aid management knowledge and practices. Such programs can be designed to target the most common sources of knowledge, such as the media and schools/universities, to ensure that the public receives accurate and current information.

In this study, the media was the most common source of information about first aid for burns (56.9%). This emphasizes the significance of utilizing various media channels to increase community awareness and education about burn first-aid management. As they are extensively utilized and accessible to a substantial portion of the population, social media platforms may be particularly useful for spreading information and increasing knowledge regarding burn first-aid management and other medical knowledge [14,15].

The most common cause of burns, according to the survey, was hot water (28.3%), followed by hot surfaces (19.9%), and combustible substances (10.1%). Multiple studies have examined the prevalence and causes of burns in various populations. Not similar to the findings of our survey, the retrospective cohort study by Akkam et al. in Saudi Arabia indicated that the majority of burns occurred at home and were caused by open fires [16,17]. Combustible chemicals were the third most prevalent cause of burns. In the study conducted by ALmultaq et al. in Saudi Arabia, for instance, scald and flame injuries accounted for the majority of burns among the general population, with hot water being the predominant source of scald injuries [16]. This is consistent with the survey’s finding that hot water is the leading cause of burns. Similarly, Alajmi et al in a cross-sectional study conducted in Saudi Arabia indicated that hot water injuries were the most common cause of burns, corroborating the findings of this survey [6]. In a previous systematic review, the authors reported that scald injuries were the most prevalent, followed by flame-related burns, which is consistent with the survey results [5]. These results indicate that focused actions are necessary to prevent and treat burns caused by these sources. For instance, steps can be taken to ensure that hot water is stored at an appropriate temperature and that children are supervised when using hot water. To prevent unintentional burns, warning labels and safety instructions can be added to hot surfaces and flammable chemicals.

A considerable minority of participants (18.5%) were unaware of the severity of their burns, according to the findings of the study. This emphasizes the need for education and awareness programs to assist individuals in identifying the various degrees of burns and understanding the appropriate first-aid measures for each degree. When individuals are unsure of the severity of their burn or if they require medical assistance, they must immediately consult a physician [18].

A significant proportion of the participants (10.7%) did not know about the best method for administering burn first aid, while others reported using traditional treatments such as applying honey or toothpaste. These traditional treatments are not advised because they may cause additional skin damage and delay proper medical care [1,19]. Burns should be irrigated with tap water for at least 10 minutes as a first-aid measure [20,21]. Therefore, education and awareness-raising activities should focus on dispelling myths and misunderstandings concerning burn first-aid management and promoting the adoption of evidence-based practices.

Regarding the significance of covering the burn, just 37% of interviewees indicated that it is essential. Covering a burn may help prevent infection and promote healing, but it is essential to use clean, sterile materials and to avoid covering it too tightly [22,23]. Education and awareness-raising campaigns should emphasize the significance of using clean and sterile materials when covering a burn and encourage individuals who are uncertain about the right first-aid measures to seek medical help.

Limitations of the study

Because of the study’s use of convenience sampling, the results may not be representative of the Al-Baha region’s population as a whole. This can provide skewed findings and hinder the study's capacity to be extrapolated to the public at large. Self-reported information was also included, which raises the possibility of recall bias, that is, the possibility that study participants exaggerated or omitted certain aspects of their background knowledge or experiences. This can reduce the reliability of the data and the overall strength of the study’s conclusions. Finally, the cross-sectional design of the study meant that it could only provide a single, static picture of the participants’ attitudes and behaviors regarding providing first aid for burns. To fully grasp the ways in which this knowledge and practice evolve over time, longitudinal research may be necessary. The authors are aware of these caveats and advise future research to improve the validity and generalizability of their findings by employing stricter sample procedures and more comprehensive data collection methodologies.

## Conclusions

This study emphasizes the necessity for targeted education and awareness-raising activities to improve the community’s understanding and habits regarding burn first-aid management. Such programs might be tailored to target the most common knowledge sources, such as the media and school/university, and debunk myths and misunderstandings concerning burn first-aid management. It is possible to reduce the outcomes and severity of burns in the community and improve health outcomes for all by enhancing the knowledge and practices of burn first-aid management.
